# Intra-abdominal hypertension among medical septic patients associated with worsening kidney outcomes (IAH-WK study)

**DOI:** 10.1097/MD.0000000000032807

**Published:** 2023-01-27

**Authors:** Nitcha Suphatheerawatr, Solos Jaturapisanukul, Surazee Prommool, Sathit Kurathong, Wanjak Pongsittisak

**Affiliations:** a Department of Internal Medicine, Faculty of Medicine Vajira Hospital, Navamindradhiraj University, Bangkok, Thailand; b Nephrology and Renal Replacement Therapy division, Department of Internal Medicine, Faculty of Medicine Vajira Hospital, Navamindradhiraj University, Bangkok, Thailand; c Vajira Renal-Rheumatology-Autoimmune Disease Research Group, Bangkok, Thailand.

**Keywords:** acute kidney injury, intra-abdominal hypertension, intra-abdominal pressure, kidney outcome, medical septic patient, sepsis

## Abstract

High intra-abdominal pressure (IAP) is associated with acute kidney injury (AKI). However, the relationship between intra-abdominal hypertension (IAH) and AKI in medical septic patients is still inconclusive. This prospective cohort study enrolled patients admitted in the Medical Intensive Care Unit from April 2020 to February 2021. Demographic, therapeutic, and laboratory data were obtained upon admission. The evaluation of IAP was performed via the intra-vesical method during the first and second 24 hours of admission. Kidney function was evaluated on the first 3 days and at least on the 7^th^ day of enrollment. Among 79 patients, 30 (38%) developed IAH, while 50 (63.3%) developed AKI within 7 days. On the first day, the mean IAP was 15.4 (interquartile range [IQR], 4) and 7.0 (IQR, 3.7) mm Hg in the IAH and non-IAH groups, respectively. A total of 52 patients (65.8%) developed the primary outcome (i.e., a composite outcome including AKI, treatment with kidney replacement therapy, or death). On Cox proportional-hazards model between IAH and outcomes, after adjustment for multiple covariates, IAH was associated with a composite outcome (hazard ratio [HR], 6.5; 95% confidence interval [CI], 2.3–18.6; *P* < .005) and the development of AKI (HR, 6.5; 95% CI, 2.3–18.8; *P* < .005). IAH was associated with a composite outcome of AKI, treatment with kidney replacement therapy, or death in medical septic patients. thaiclinicaltrial.org, Identifier: TCTR20200531001, Registered May 24, 2020.

## 1. Introduction

Intra-abdominal hypertension (IAH) is defined by an intra-abdominal pressure (IAP) > 12 mm Hg, whereas abdominal compartment syndrome is defined by an IAP > 20 mm Hg with new organ dysfunction.^[1]^ For most critically ill patients, an IAP of 5 to 7 mm Hg is considered normal. The risk factors leading to IAH include primary disease in the abdominopelvic region (e.g., severe pancreatitis, abdominal surgery, and intraperitoneal hemorrhage), while secondary causes include conditions that do not originate from the abdominopelvic region (i.e., severe burn or septic shock). Multiple factors have been associated with increased IAP in sepsis, such as increased bowel wall permeability, bowel ileus, and aggressive intravenous hydration.^[1,2]^

IAH is associated with morbidity and mortality in critically ill patients, and may even cause multiorgan dysfunction. Early detection is necessary to prevent the complications caused by IAH. Previous studies reported that the prevalence of IAH among critically ill patients ranges from 30% to 85%, which varies across clinical settings (i.e., surgical or medical).^[3–9]^

A previous report on the relationship between IAP and acute kidney injury (AKI) in postoperative abdominal surgery showed that the incidence of IAH was 26.67%. Moreover, an IAP of > or equal to 7.68 mm Hg was a predictor of AKI with a sensitivity of 87% and specificity of 46%.^[10]^ However, association between IAH and AKI in medical septic patients remains inconclusive. This study aimed to evaluate the incidence of IAH and the relationship between IAH and kidney outcomes among medical septic patients.

## 2. Methods

### 2.1. Study design

This prospective cohort study enrolled patients admitted at the medical intensive care unit (ICU) from April 2020 to February 2021 in a university hospital in Thailand. We early stopped the study because of the pandemic of coronavirus infectious disease 2019, which resulted in the grant being terminated.

### 2.2. Study population

We included patients aged over 18 years, diagnosed with sepsis or septic shock, and admitted at the medical ICU. The exclusion criteria were as follows: chronic kidney disease (CKD) stage 4 or greater, pregnancy, contraindicated or denied for Foley catheter insertion, end-stage cancer, acute kidney injury stage 3 (acute kidney injury network [AKIN] stage 3) at admission, and death within 24 hours after enrollment.

### 2.3. Data collection

The following data were collected upon admission: gender, age, preexisting comorbidities, current medications, baseline hemoglobin and creatinine, date of hospital and ICU admission, and source of infection. The following clinical characteristics were also recorded: body mass index (BMI), acute physiological and chronic health evaluation II (APACHE II) score, presence of systemic inflammatory response syndrome, presence of septic shock, and glasgow coma scale score. systemic inflammatory response syndrome was defined as fulfilling 2 out of the following 4 criteria: the body temperature > 38 °C or < 36 °C, heart rate > 90 bpm, respiratory rate > 20 per minute or arterial pressure of carbon dioxide < 32 mm Hg, and white blood cell count > 12,000 or < 4000 cells/mm^3^.^[11]^ Sepsis was defined as life-threatening organ dysfunction caused by a dysregulated host response to infection (sepsis-3).^[11]^ We defined medical sepsis as patients diagnosed with sepsis criteria and hospitalized in the medical ICU/semi-ICU with not being required immediate or urgent surgery. Septic shock was defined having a clinical picture of sepsis with persistent hypotension requiring vasopressors to maintain mean arterial blood pressure ≥ 65 mm Hg and serum lactate level > 2 mmol/L despite adequate volume resuscitation.^[11]^ However, serum lactate is not the routine practice laboratory in our hospital. After collecting data, many patients clinically diagnosed with septic shock did not have lactate levels. Hence, the definition of septic shock was modified to sepsis with persistent hypotension requiring vasopressors to maintain mean arterial blood pressure ≥ 65 mm Hg and clinically diagnosed by the attending physician. We recorded the following information: need for vasopressors, need for diuretics, and use of nephrotoxic agents (i.e., aminoglycoside, vancomycin, and colistin), exposure to contrast media, use of sedative drugs, use of corticosteroids, fluid correction therapy, blood transfusion, laboratory data, and ventilator settings. The following data were recorded within 7 days after enrollment: kidney function, treatment with kidney replacement therapy (KRT), and death. Kidney function was evaluated on the first 3 days and at least on the 7^th^ day of enrollment.

### 2.4. IAP measurement

The initial IAP measurement was taken twice, specifically during the first and second 24 hours of admission to ICU/semi-ICU. IAP was measured using the intra-vesical method, described as follows. First, the patient was placed completely supine. Second, while maintaining aseptic technique, the Foley catheter was clamped to a urine bag, and a No.24 needle was inserted into the urinary port. The zero-reference point of the transducer is placed at the level of the mid-axillary line. Then, 25 mL of sterile water was infused into the bladder, and the IAP was measured at end-expiration, 1 minute after infusion. The IAP expressed in cmH_2_O was converted to mm Hg by multiplied it by 0.73. After evaluation, the Foley catheter was unclamped, and the infused volume was subtracted from the urinary volume output recorded at that time. Patients with an IAP > 12 mm Hg were considered to have IAH. abdominal compartment syndrome was defined as having an IAP > 20 mm Hg with new organ dysfunction.^[1]^

### 2.5. Outcomes

The primary outcome was an association between IAH and a composite outcome including AKI, treatment with KRT, or death within 7 days. AKI was defined as an increased baseline creatinine ≥ 0.3 mg/dL within 48 hours or increasing by ≥ 1.5 times within 7 days.^[12]^ The secondary outcomes were the incidence of IAH, the association between IAH and individual outcomes of AKI, KRT, and death.

### 2.6. Statistical analysis

We calculated the required sample size for a 2-sided level of significance of α = 0.05 and power of 80% with a dropout rate of 15%. We found that at least 94 patients (≥25 patients with IAH and ≥ 69 patients with normal IAP) were required to produce a significant difference. The difference was estimated from a previous study in which the proportion of AKI was 68.8% in the IAH group and 34.1% in without IAH group.^[10]^

Continuous variables were expressed as mean ± standard deviation for normal distribution and as median with interquartile range (IQR) for non-normal distribution. T-test and Mann–Whitney *U* test were used to compare the statistical differences in the mean and median between 2 groups, respectively. The normal distribution was tested by the Shapiro–Wilk test. Categorical variables were expressed as their number and percentage. Chi-squared test was performed to identify significant differences between categorical data. The time to a composite outcome, AKI, and death was plotted with a Kaplan–Meier curve and analyzed using log-rank test. The Cox proportional-hazards model was used to analyze the hazard ratio between IAH and the aforementioned outcomes (i.e., composite outcome, AKI, and death). Two models with certain adjustments were made. Model 1 was adjusted for age, gender, BMI, and comorbidities (e.g., diabetes mellitus, hypertension, cirrhosis, history of cerebrovascular accident, atherosclerotic cardiovascular disease, chronic obstructive pulmonary disease, CKD, and dyslipidemia). On the other hand, model 2 was adjusted similarly to model 1, but also included baseline serum creatinine, hemoglobin, APACHE II score, presence of septic shock, need for vasopressors, need for mechanical ventilator supports, need for diuretics, use of nephrotoxic drugs, exposure to contrast media, and use of blood transfusion. We performed post hoc analysis by specified patients with septic shock to analyze the association between IAH and outcomes. A *P* < .050 was used as a threshold for statistical significance. Statistical analysis was performed using Python version 3.7.10 (library-package: Pandas, 1.1.5; NumPy, 1.19.5; Stats models, 0.11.1; and Lifelines, 0.25.8).

## 3. Results

**Figure 1.**
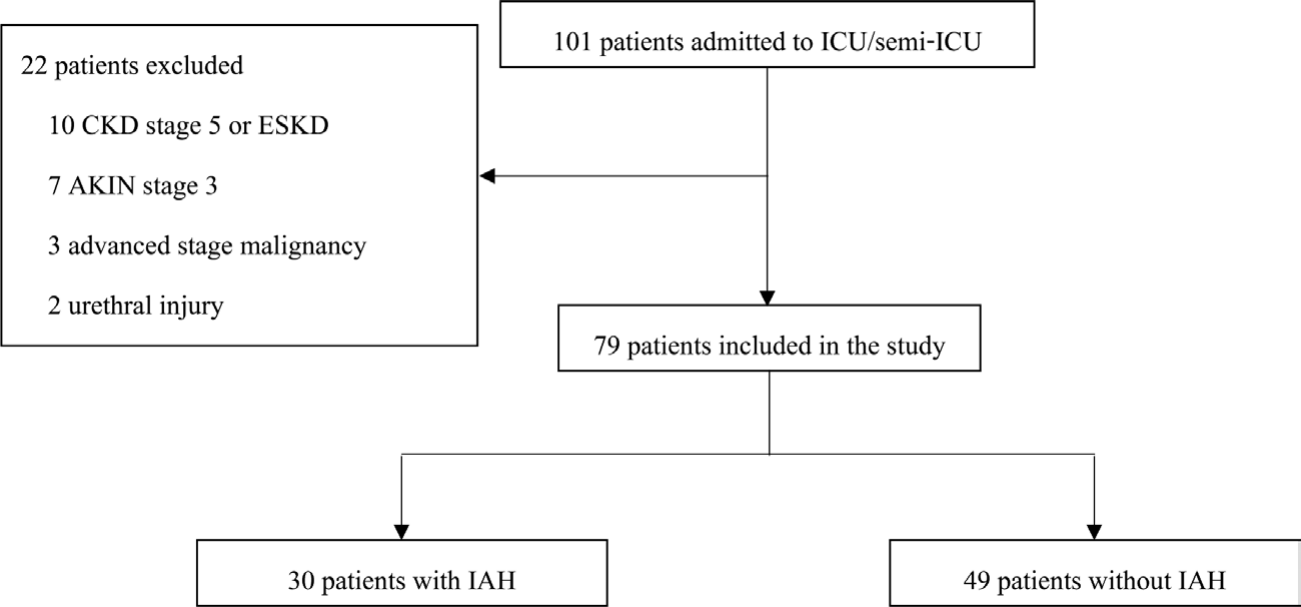
Flowchart of participant enrollment. AKIN = acute kidney injury network, CKD = chronic kidney disease, ESKD = end-stage kidney disease, IAH = intra-abdominal hypertension, ICU = intensive care unit.

The study was conducted from April 2020 to February 2021. A total of 101 patients were admitted during the study period (Fig. 1), but 22 (22.3%) patients were excluded due to CKD stage 5 or end-stage kidney disease on KRT (10 patients), AKIN stage 3 (7 patients), advanced stage malignancy (3 patients), and urethral injury (2 patients). The 79 remaining patients were included in the study.

### 3.1. Baseline characteristics

The mean age was 63.2 years, and 53 (67.1%) patients were male. Patients who developed IAH, compared to those without IAH, had significantly higher BMI, serum creatinine at admission, proportion of patients with contrast media exposure, proportion of patients who received vancomycin, patients who required sedative drugs, proportion of patients who needed a mechanical ventilator, and higher positive end-expiratory pressure /peak airway pressure (Ppeak). On the other hand, patients without IAH had significantly more cerebrovascular accidents, higher platelet count, and serum albumin. The APACHE II score at admission had a mean value of 22.4 ± 7.1. Septic shock was found in 54 (68.4%) patients (Table 1).

**Table 1:** Baseline characteristics of critically septic patients*.

Characteristic	Overall (N = 79)	IAH group (n = 30)	No-IAH group (n = 49)	*P* value
Age (yr)	63.2 ± 15.2	59.3 ± 14.1	65.6 ± 15.4	.080
Male sex no. (%)	53 (67.1)	22 (73.3)	31 (63.3)	.542
BMI†	22.7 ± 4.6	25.2 ± 3.7	21.1 ± 4.4	<.001
Underlying disease no. (%)				
DM	29 (36.7)	8 (26.7)	21 (42.9)	.147
HT	40 (50.6)	15 (50.0)	25 (51.0)	.930
DLP	19 (24.1)	5 (16.7)	14 (28.6)	.230
CVA	13 (17.6)	1 (3.3)	12 (24.5)	.014
Cirrhosis	9 (11.4)	5 (16.7)	4 (8.2)	.248
CAD	4 (5.1)	3 (10.0)	1 (2.0)	.117
COPD	4 (5.1)	2 (6.7)	2 (4.1)	.611
Baseline CKD				.511
CKD stage 1–2	2 (2.5)	1 (3.3)	1 (2.0)	
CKD stage 3	6 (7.6)	1 (3.3)	5 (10.2)	
Medication no. (%)				
ACEI/ARB	15 (19.0)	3 (10.0)	12 (24.5)	.111
NSAIDs	2 (2.5)	2 (6.7)	0 (0.0)	.161
Diuretic	12 (15.2)	6 (20.0)	6 (12.3)	.351
Principle diagnosis no. (%)				
Pulmonary infectionǂ	38 (48.2)	12 (40.1)	26 (53.0)	
Hepatobiliary infection‖	2 (2.5)	2 (6.6)	0 (0.0)	
GI tract infection‡	8 (10.1)	2 (6.6)	6 (12.2)	
GU tract infection§	4 (5.0)	0 (0.0)	4 (8.2)	
Primary septicemia	21 (26.6)	12 (40.1)	9 (18.4)	
Other¶	6 (7.6)	2 (6.6)	4 (8.2)	
Laboratory baseline				
Hemoglobin g/dL	11.2 ± 2.2	11.3 ± 2.5	11.1 ± 2.1	.815
Creatinine mg/dL	1.00 (0.43)	1.00 (0.45)	1.00 (0.41)	.373
APACHE II	22.4 ± 7.1	24.3 ± 7.2	21.3 ± 6.6	.066
Septic shock no. (%)	54 (68.4)	17 (56.7)	37 (75.5)	.081
Laboratory day 1				
Hemoglobin g/dL	10.5 ± 2.4	10.6 ± 2.2	10.5 ± 2.5	.896
WBC cells/mm^3^	12,150 (7700)	14,100 (12,067)	11,920 (6290)	.412
Platelet × 10^3^ cells/mm^3^	145.5 (213)	94.5 (91.5)	231 (204)	.007
BUN mg/dl	29 (27.0)	29 (19.8)	30 (25.0)	.241
Creatinine mg/dL	1.34 (1.2)	1.53 (1.1)	1.22 (1.1)	.034
Sodium mmol/L	134 (8)	135 (6)	134 (8)	.500
Potassium mmol/L	4.10 (0.9)	3.99 (0.7)	4.21 (1.0)	.474
Albumin g/dL	2.8 (1.3)	2.4 (0.9)	3.0 (1.6)	.014
pH	7.39 (0.08)	7.34 (0.10)	7.39 (0.06)	.038
Medication used no. (%)				
Vasopressor	56 (70.9)	18 (60.0)	38 (77.6)	.096
Diuretic	36 (45.6)	13 (43.3)	23 (46.9)	.755
Colistin	4 (2.7)	3 (10.0)	1 (2.0)	.117
Vancomycin	13 (16.5)	10 (33.3)	3 (6.1)	.002
Sedation	51 (62.2)	24 (80.0)	27 (55.1)	.025
Corticosteroid	35 (44.3)	12 (32.0)	23 (46.9)	.547
Contrast exposure no. (%)	18 (22.8)	11 (36.7)	7 (14.3)	.021
Blood transfusion no. (%)	36 (45.6)	16 (53.3)	20 (40.8)	.278
Mechanical ventilator no. (%)	68 (86.1)	29 (96.7)	39 (79.6)	.033
Ventilator				
PEEP mm Hg	5 (1)	5 (2)	5 (0)	.036
Ppeak mm Hg	21.0 (3.0)	22.0 (4.0)	20.0 (2.3)	.002
IAP mm Hg				
Day 1	10.3 (7.4)	15.4 (4.0)	7.0 (3.7)	<.001
Day 2	10.4 (8.0)	14.7 (4.6)	7.0 (5.5)	<.001
APP mm Hg				
Day 1	67.9 (17.9)	63.6 (22.0)	69.7 (16.3)	.016
Day 2	71.3 (19.6)	68.0 (13.5)	73.0 (20.5)	.039
Cumulative fluid mL¥	2325 (2620)	3300 (1800)	2000 (1600)	.088

ACEI = angiotensin converting enzyme inhibitor, APACHE II = acute physiology and chronic health evaluation II, APP = abdominal perfusion pressure, ARB = angiotensin receptor blocker, BUN = blood urea nitrogen, CAD = coronary artery disease, CKD = chronic kidney disease, COPD = chronic obstructive pulmonary disease, CVA = cerebrovascular accident, DLP = dyslipidemia, DM = diabetes mellitus, GI = gastrointestinal, GU = genitourinary, HT = hypertension, IAH = intra-abdominal hypertension, IAP = intra-abdominal pressure, IQR = interquartile range, ICU = intensive care unit, NSAID = nonsteroidal anti-inflammatory drugs, PEEP = positive end-expiratory pressure, Ppeak = peak airway pressure, WBC = white blood cell count.

* Plus-minus values indicate means ± SD. For continuous data, parentheses with number indicate median (IQR).

† BMI = body mass index is the weight in kg divided by height in m^2^.

ǂ Pulmonary infection includes empyema thoracis, lung abscess, pneumonia, and aspiration pneumonia.

‖ Hepatobiliary infection includes empyema cholecystitis and cholangitis.

‡ GI tract infection includes intra-abdominal infection, spontaneous bacterial peritonitis, infective diarrhea, and perianal abscess.

§ GU tract infection includes urinary tract infection.

¶ Other includes infective endocarditis and central nervous system infection.

¥ Cumulative fluid is total fluid intake before admission to ICU/semi-ICU.

The most common infection source was pulmonary infection (38 patients, 48.2%), which included cases of empyema thoracis, lung abscess, pneumonia, and aspiration pneumonia. The second most common source was primary septicemia (21 patients, 26.6%). The mean volume of fluid received upon admission was 2325 mL on the first day, higher in the IAH group than the no-IAH group, 3300 and 2000 mL, respectively; however, there was no significance (*P* = .088).

A total of 30 (37.9%) patients developed IAH. The mean IAP was higher among patients with IAH versus those without IAH (15.4 vs 7.0 mm Hg, *P* < .01). Abdominal perfusion pressure was also significantly higher among those without IAH.

### 3.2. Outcomes

There were 52 (65.8%) patients who developed a composite outcome. Regarding individual outcomes, AKI, KRT, and death were seen in 50 (63.1%), 5 (6.3%) patients, and 10 (12.7%) patients, respectively. Composite outcomes were not statistically different between both groups. KRT was significantly more frequent among those with IAH compared to those without (Table 2). The incidence rate of AKI was 0.329 events per patient-day and 0.109 events per patient-day for IAH and non-IAH groups, respectively. The maximum staging of AKI was significantly higher (*P* = .007) among those with IAH versus those without. AKIN stage 1, 2, and 3, respectively, were seen in 4 (16.0%), 4 (16.0%), and 10 (40.0%) patients with IAH, and in 14 (28.6%), 9 (18.4%), and 5 (10.2%) patients without IAH. Furthermore, the IAH group developed AKI and died significantly earlier than those without IAH.

**Table 2:** Association between intra-abdominal hypertension and outcomes.

	Overall (N = 79)	IAH group (n = 30)	No-IAH group (n = 49)	*P* value
Composite outcome no. (%)†	52 (65.8)	23 (76.7)	29 (59.2)	.112
Secondary outcome no. (%)				
AKI	50 (63.3)	22 (73.3)	28 (57.1)	.147
KRT	5 (6.3)	4 (13.3)	1 (2.0)	.045
Death	10 (12.7)	5 (16.7)	5 (10.2)	.402
Incidence rate of AKI event/patient-d		0.329	0.109	NA
Median time to outcome (IQR) d				
Time to composite outcome†	1.0 (1.0)	1.0 (1.0)	2.0 (2.0)	.039
Time to AKI	0.0 (1.0)	0.0 (0.8)	0.5 (2.0)	.025
Time to KRT	3.0 (2.0)	2.5 (1.5)	4.0 (0.0)	.234
Time to death	3.5 (2.5)	2.0 (1.0)	5.0 (1.0)	.016
Maximum stage of AKI no. (%)				.007
Stage I	18 (36.0)	4 (18.2)	14 (50.0)	
Stage II	14 (28.0)	5 (22.7)	9 (32.1)	
Stage III	18 (36.0)	13 (59.1)	5 (17.9)	

AKI = acute kidney injury, IAH = intra-abdominal hypertension, IQR = interquartile range, KRT = kidney replacement therapy, NA = not applicable.

† Composite outcome was AKI, KRT, and death.

### 3.3. Time-to-event analysis

**Figure 2.**
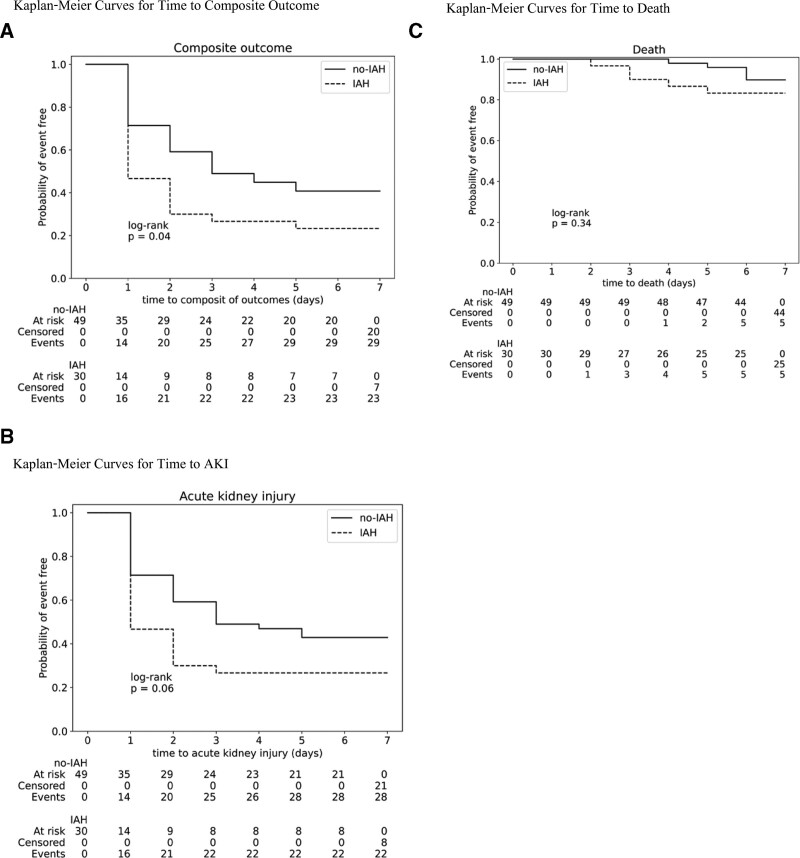
Probability of event-free. Panel A shows the Kaplan–Meier curves for the first event of the composite outcome, defined as the occurrence of AKI, treatment with KRT, or death. Panel B shows the Kaplan–Meier curves for time to the first event of AKI. Panel C shows the Kalan-Meier curves for time to death. AKI = acute kidney injury, IAH = intra-abdominal hypertension, KRT = kidney replacement therapy.

The Kaplan–Meier curves of events and both groups are shown in Figure 2. In the Cox proportional-hazards model between IAH and outcomes, IAH was an independent predictor for a composite outcome (HR, 1.81; 95% CI, 1.04–3.14; *P* = .030) and also AKI but not death (Table 3). After adjusting for multiple covariates (Model 2), IAH was significantly associated with the development of a composite outcome (HR, 6.58; 95% CI, 2.33–18.58; *P* < .005) and AKI (HR, 6.52; 95% CI, 2.26–18.79; *P* < .005).

**Table 3:** Cox proportional hazard model of intra-abdominal hypertension and outcomes.

	Crude		Model 1†		Model 2‡	
	HR (95% CI)	*P* value	HR (95% CI)	*P* value	HR (95% CI)	*P* value
Composite outcome*	1.81 (1.04–3.14)	.030	1.72 (0.81–3.64)	.160	6.58 (2.33–18.58)	<.005
AKI	1.78 (1.01–3.12)	.040	1.72 (0.81–3.67)	.160	6.52 (2.26–18.79)	<.005
Death	1.79 (0.52–6.20)	.360	0.98 (0.18–5.22)	.980	1.80 (0.02–151.99)	.800

ACEI = angiotensin converting enzyme inhibitor, AKI = acute kidney injury, APACHE II = = acute physiology and chronic health evaluation II, ARB = angiotensin receptor blocker, BMI = body mass index, CAD = coronary artery disease, CKD = chronic kidney disease, COPD = chronic obstructive pulmonary disease, Cr = creatinine, CVA = cerebrovascular accident, DLP = dyslipidemia, DM = diabetes mellitus, Hb = hemoglobin, HT = hypertension, NSAIDs = nonsteroidal anti-inflammatory drugs.

* Composite outcome included AKI, KRT, or death.

† Model 1: adjusted for age, gender, BMI, comorbid (DM, HT, cirrhosis, old CVA, CAD, COPD, CKD, and DLP), previous medications (ACEI, ARB, NSAIDs, and diuretic).

‡ Model 2: adjusted for model 1 additional with baseline Cr, baseline Hb, APACHE II, presented with septic shock, required vasopressor, required diuretic, current medication (colistin, vancomycin, sedative agents, and corticosteroid), received blood transfusion, need mechanical ventilator, exposed to contrast media.

### 3.4. Post Hoc analysis

Seventeen patients from overall septic shock (N = 54) had IAH. AKI occurred in 14/17 (82.4%) in the IAH group and 22/37 (59.5%) in the non-IAH group with no statistical difference. The composite outcome, KRT, and death were no statistical differences between groups (Table 4).

**Table 4:** Association between intra-abdominal hypertension and outcomes in septic shock patients.

	IAH group (n = 17)	No-IAH group (n = 37)	Odds ratio (95% CI)	*P* value
Composite outcome no. (%)†	14 (82.4)	23 (62.2)	2.84 (0.69–11.67)	.138
Secondary outcome no. (%)				
AKI	14 (82.4)	22 (59.5)	3.18 (0.78–13.02)	.097
KRT	2 (11.8)	1 (2.7)	4.80 (0.40–57.02)	.177
Death	3 (17.7)	5 (13.5)	1.37 (0.29–6.55)	.691

AKI = acute kidney injury, KRT = kidney replacement therapy, IAH = intra-abdominal hypertension.

† Composite outcome was AKI, KRT, and death.

## 4. Discussion

The interest in IAH has risen because of its effect on multiorgan dysfunction and association with increased mortality, and thus its early detection is important to prevent these complications. There are multiple pathways through which IAH develops in septic patients. In this study, we aimed to demonstrate the association between IAH and worsening kidney outcomes among medical septic patients. IAH had an incidence of 38% (n = 30/79), among which 23 patients (76.7%) developed a composite outcome, while 22 patients (73.3%) developed AKI. Cox proportional-hazards model showed that a diagnosis of IAH among medical septic patients upon admission was an independent predictor for AKI, KRT, and death.

Our study shows that IAH was associated with high BMI, low platelet count, low serum albumin, and need for ventilator use with high peak pressure; these findings are in line with the results of a previous large multicenter cohort study.^[13]^ Another previous study demonstrated that septic patients who developed IAH had a mortality rate of 50%, in contrast to 19.4% among septic patients without IAH (*P* < .001).^[14]^ Moreover, another study of septic shock patients admitted to a surgical-medical ICU found that the coexistence of septic shock and IAH was associated with more severe kidney dysfunction compared to septic shock without IAH.^[6]^ Previous clinical studies showed that IAH was associated with worsening kidney outcomes in various situations.^[10,15,16]^ The meta-analysis recently confirmed that IAH increases AKI risk.^[17]^ IAH caused reduction in arterial inflow and venous outflow from kidney, then reduction in renal perfusion had developed. Reduction in glomerular hydrostatic pressure from renal hypoperfusion additional with increased Bowman space hydrostatic pressure from intraabdominal hypertension may result in reduction in glomerular filtration gradient which may be the cause of AKI. The treatment options for reduction of IAP in patients with sepsis are reduction of intraluminal contents by nasogastric suction to decompress intragastric content, laxative or rectal tube insertion to decompress intracolonic content and prescription of prokinetic agents, reduction of intra-abdominal fluid collection by abdominal paracentesis to release ascites in case of massive ascites or surgical evacuation of the lesion, improvement of abdominal wall compliance by adequate sedation, analgesia and neuromuscular blockade, optimization of fluid administration by avoid excessive fluid resuscitation, consider diuretic or even ultrafiltration in case of fluid overload, optimization of systemic and regional perfusion.^[18]^

The strengths of this study include its prospective study design and its novelty as the first study to our knowledge which describes the association between IAH and kidney outcomes in medical septic patients. On the other hand, the limitation of this study is: short follow-up period, in which long-term consequences were not evaluated; we did not perform the standard technique for IAH measurement (due to limited resource setting); this study did not have data of typical markers for sepsis (e.g., lactate, procalcitonin); and the overall sample size was not completed; however, the IAH patients of our study achieved the target of sample size. Hence, we recalculated the power of the study by this actual sample size. The power of the study is about 80%, equivalent to pre-specified power because the estimated sample size was calculated with a 15% dropout rate.

## 5. Conclusions

IAH was associated with the development of a composite outcome including AKI, treatment with KRT, and death in critically septic patients.

## Acknowledgments

We greatly appreciate the assistance provided by Medical ICU and semi-ICU service teams of Vajira Hospital.

## Author contributions

**Conceptualization:** Nitcha Suphatheerawatr, Surazee Prommool, Sathit Kurathong, Wanjak Pongsittisak.

**Data curation:** Nitcha Suphatheerawatr.

**Formal analysis:** Nitcha Suphatheerawatr, Wanjak Pongsittisak.

**Investigation:** Nitcha Suphatheerawatr, Wanjak Pongsittisak.

**Methodology:** Nitcha Suphatheerawatr, Solos Jaturapisanukul, Wanjak Pongsittisak.

**Project administration:** Nitcha Suphatheerawatr, Sathit Kurathong, Wanjak Pongsittisak.

**Resources:** Wanjak Pongsittisak.

**Funding acquisition:** Sathit Kurathong, Wanjak Pongsittisak.

**Supervision:** Surazee Prommool, Sathit Kurathong.

**Validation:** Nitcha Suphatheerawatr, Solos Jaturapisanukul.

**Writing – original draft:** Nitcha Suphatheerawatr.

**Writing – review & editing:** Solos Jaturapisanukul, Surazee Prommool, Sathit Kurathong, Wanjak Pongsittisak.
